# Identification of *CHIP* as a Novel Causative Gene for Autosomal Recessive Cerebellar Ataxia

**DOI:** 10.1371/journal.pone.0081884

**Published:** 2013-12-02

**Authors:** Yuting Shi, Junling Wang, Jia-Da Li, Haigang Ren, Wenjuan Guan, Miao He, Weiqian Yan, Ying Zhou, Zhengmao Hu, Jianguo Zhang, Jingjing Xiao, Zheng Su, Meizhi Dai, Jun Wang, Hong Jiang, Jifeng Guo, Yafang Zhou, Fufeng Zhang, Nan Li, Juan Du, Qian Xu, Yacen Hu, Qian Pan, Lu Shen, Guanghui Wang, Kun Xia, Zhuohua Zhang, Beisha Tang

**Affiliations:** 1 Department of Neurology, Xiangya Hospital, Central South University, Changsha, China; 2 The State Key Laboratory of Medical Genetics, Changsha, China; 3 BGI-Shenzhen, Shenzhen, China; 4 Department of Pharmacology, College of Pharmaceutical Sciences, Soochow University, Suzhou, China; 5 The Key Laboratory of Hunan Province in Neurodegenerative Disorders, Central South University, Changsha, China; 6 T-Life Research Center, Fudan University, Shanghai, China; 7 Department of Biology, University of Copenhagen, Copenhagen, Denmark; 8 King Abdulaziz University, Jeddah, Saudi Arabia; 9 The Novo Nordisk Foundation Center for Basic Metabolic Research, University of Copenhagen, Copenhagen, Denmark; 10 Centre for iSequencing, Aarhus University, Aarhus C, Denmark; Emory University, United States of America

## Abstract

Autosomal recessive cerebellar ataxias are a group of neurodegenerative disorders that are characterized by complex clinical and genetic heterogeneity. Although more than 20 disease-causing genes have been identified, many patients are still currently without a molecular diagnosis. In a two-generation autosomal recessive cerebellar ataxia family, we mapped a linkage to a minimal candidate region on chromosome 16p13.3 flanked by single-nucleotide polymorphism markers rs11248850 and rs1218762. By combining the defined linkage region with the whole-exome sequencing results, we identified a homozygous mutation (c.493CT) in *CHIP* (NM_005861) in this family. Using Sanger sequencing, we also identified two compound heterozygous mutations (c.389AT/c.441GT; c.621C>G/c.707GC) in *CHIP* gene in two additional kindreds. These mutations co-segregated exactly with the disease in these families and were not observed in 500 control subjects with matched ancestry. CHIP colocalized with NR2A, a subunit of the N-methyl-D-aspartate receptor, in the cerebellum, pons, medulla oblongata, hippocampus and cerebral cortex. Wild-type, but not disease-associated mutant CHIPs promoted the degradation of NR2A, which may underlie the pathogenesis of ataxia. In conclusion, using a combination of whole-exome sequencing and linkage analysis, we identified *CHIP*, encoding a U-box containing ubiquitin E3 ligase, as a novel causative gene for autosomal recessive cerebellar ataxia.

## Introduction

Autosomal recessive cerebellar ataxias (ARCAs) are a group of progressive neurodegenerative disorders with marked clinical and genetic heterogeneity. ARCAs are characterized by gait impairment, poor balance, frequent falls, disturbed limb coordination, dysarthria and eye movement abnormalities, and these characteristics are most likely due to the degeneration of cerebellum, brainstem and spinal cord [1-5]. Patients with ARCAs often present with diverse neurological and extraneurological symptoms (e.g., bone malformations, cardiomyopathies and cataracts). ARCAs can be grouped into five major classes, that include mitochondrial energy deficiencies (e.g., Friedreich’s ataxia), ataxias with DNA repair defects (e.g., ataxia telangiectasia), metabolic ataxias (e.g., ataxia with vitamin E deficiencies), congenital ataxias (e.g., Joubert ataxia) and degenerative ataxias (e.g., spastic ataxia of Charlevoix Saguenay) [1-9]. Although more than 20 disease-causing genes have been identified in ARCAs, many patients with ARCAs are currently without a molecular diagnosis [4,5,10-12]. 

Whole-exome sequencing provides a powerful and affordable means to identify Mendelian disease-causing genes, especially for diseases with great high genetic or clinical heterogeneity. We used the combined strategy of whole-exome sequencing and linkage analysis, to identify transglutaminase 6 as a novel causative gene of autosomal dominant spinocerebellar ataxias in 2010 and proline-rich transmembrane protein 2 as a causative gene of paroxysmal kinesigenic dyskinesias in 2011 [13,14]. Here, we applied the same strategy to screen for the causative gene of ARCA. 

## Materials and Methods

### Subjects

The clinical data and blood samples were obtained from three Han Chineses families with histories of ARCA that included 6 affected individuals. A cohort of 36 additional unrelated ARCA pedigrees and 196 sporadic ataxia patients were chosen for subsequent mutational screening analyses. These analyses included gene mutation screenings for, and exclusion of subjects with the following: Friedreich's ataxia, ataxia with vitamin E deficiency, ataxia plus oculomotor apraxia type 1, ataxia plus oculomotor apraxia type 2 and so on. We also screened for, and excluded subjects with the following more frequent subtypes of autosomal dominant cerebellar ataxia: nucleotide expansion mutations of the *ATXN1*, *ATXN2*, *ATXN3*, *CACNA1A*, *ATXN7*, *ATXN8OS*, *ATXN10*, *PPP2R2B*, *TBP*, *NOP56* and *atrophin-1* genes in the SCA1, SCA2, SCA3/MJD, SCA6, SCA7, SCA8, SCA10, SCA12, SCA17, SCA36 and DRPLA, point mutations of *KCNC3*, *PRKCG*, *KCND3*, *PDYN*, *FGF14*, *AFG3L2*, and *TGM6* gene in the SCA13, SCA14, SCA19, SCA23, SCA27, and SCA28 substypes, and insertion and deletion mutations of the *SPTBN2*, *TTBK2*, *KCND3*, *ITPR1* and *BEAN* genes in the SCA5, SCA11, SCA22, SCA15/16/29 and SCA31 subtypes. The analyses also included 500 unaffected healthy individuals who were matched for geographical ancestry as controls. Written informed consent was obtained from each subject or their guardian. This study was approved by the Ethic Committee of the Xiangya Hospital of Central South University in China (equivalent to an Institutional Review Board).

### Exome sequencing, copy number variations (CNVs), and linkage analysis.

Genomic DNA was extracted from whole peripheral blood using standard methods (QIAGEN, Valencia, CA). Qualified genomic DNA extracted from two affected individuals in family 1 (Samples II:2 and II:3) was sheared by sonication and then hybridized to the SureSelect Biotiny-lated RNA Library for enrichment according to the manufacturer’s instructions. The enriched library targeting the exome was sequenced on the HiSeq 2000 platform to acquire paired-end reads with read length of 90 base pair. The sequenced reads were aligned to the human genome reference (UCSC hg 18 version) using SOAPaligner [15]. Next, those reads that were aligned in the desired target regions were collected for SNP calling and subsequent analysis. We estimated quality scores and made the consensus SNP callings using SOAPsnp (v 1.03) (Li et al., 2010). The low quality variations were filtered out based on the following criteria: (i) consensus quality score <20; (ii) average copy number at the allele site >=2; (iii) distance of two adjacent SNPs <5 bp; and (iv) sequencing depth <4 or >500. For indels in the targeted exome regions, we aligned the reads to the reference genome using BWA (http://bio-bwa.sourceforge.net). The alignment results were used to identify the breakpoints by gatk. Finally, we annotated the genotypes of insertions and deletions [16]. We have shared our deep-sequencing data in NIH Short Read Archive and the accession number is SRA105955.

To exclude CNVs in the critical interval, whole genome CNV analyses of seven samples (I:1, I:2 and II:1-5) were performed using the Illumina HumanHap660 BeadChip. CnvPartition for GenomeStudio was used to call CNVs, and only samples with call rates > 98% were included. To localize the disease-causing gene, we also carried out linkage analyses of whole-genome SNPs. The genotype assignments were determined using GenomeStudio genotyping module software (Illumina). Two-point logarithm of odds scores were calculated using the MERLIN linkage program version 1.01 [17]. Marker allele frequencies were estimated from the founders of the pedigree via MERLIN, and the disease was considered to be autosomal recessive with a frequency of 0.0001 and a penetrance of 95%.

### Plasmids

Full-length human *CHIP* cDNA was amplified by PCR using primers W1/W2 from the human fetal brain cDNA library as the template and inserted in-frame into p3xFlag-CMV-24 (Sigma, USA) at EcoRI/SalI sites. Mutant CHIP^N130I^, CHIP^W147C^, CHIP^L165F^, CHIP^Y207X^, and CHIP^S236T^ were generated with QuikChange site-directed mutagenesis protocol (Stratagene, La Jolla, CA, USA) with M1/M2, M3/M4, M5/M6, M7/M8, and M9/M10 primers, respectively. The primers used in this study are shown in Table S1 of File S1. Full-length human NR2A cDNA and Fbx2 cDNA were artificially synthesized by the TaKaRa Biotechnology Company (Dalian, China) and cloned into the pcDNA3.1-myc-his-B(-) vector (Invitrogen, USA) at EcoR I/BamH I sites and pKH3-HA at BamH I/Xho I sites, respectively. All constructs were confirmed by sequencing. 

### Cell culture and transfections

Human Embryonic Kidney 293 cells were grown at 37°C under 5% CO2 in Dulbecco’s modified Eagle medium supplemented with 10% fetal bovine serum and 100 U/ml of penicillin/streptomycin. Expression plasmids were transfected into cells using Lipofectamine 2000 reagent (Invitrogen, Carlsbad, CA, USA) according to the manufacturer’s protocol. 

### Immunoblot analysis

Proteins were separated by 6%-10% SDS–PAGE and transferred onto polyvinylidene difluoride membranes (Millipore, USA). The following primary antibodies were used: monoclonal anti-Flag antibody (Sigma, USA); polyclonal anti-Myc antibody (Cell Signaling Technology, USA); and monoclonal anti-HA antibody (Millipore, USA). Sheep anti-rabbit antibody (Amersham Pharmacia Biotech) was used as the secondary antibody. Detection was performed using the ECL plus Western blotting detection system (GE Healthcare, USA) according to manufacturer’s instruction. Bands were scanned and quantified by densitometric analysis with the NIH ImageJ software and normalized to β-actin as a loading control.

### Mouse brain immunofluorescence

C57BL/6 mice (8-10 w) were used for these studies. After fixation in 4% phosphate-buffered paraformaldehyde, mouse brains were frozen and cut coronally into 15 μm thick slices. After pre-incubation, the slices were incubated at 4°C for 48 h with a rabbit anti-CHIP antibody (Santa Cruz, USA) then visualized with Cy3-conjugated AffiniPure Goat anti-Rabbit IgG (Jackson ImmunoResearch laboratories, USA). For the double immunofluorescence staining, the brain sections were treated with the rabbit anti-CHIP antibody and a mouse anti-NR2A monoclonal antibody (Millipore, USA). A mixture of Cy3-conjugated AffiniPure Goat Anti-Rabbit IgG and Dylight-488 conjugated AffiniPure Goat anti-Mouse IgG (Jackson ImmunoResearch laboratories, USA) was employed as a secondary antibody. Finally, images were acquired with a a confocal laser microscope (Leica, Germany). All procedures regarding the care and use of animals were approved by the Institutional Animal Care and Use Committee of Central South University of China, and all methods used in the experiments were in accordance with institutional regulations and the guidelines of the Hunan Animal Care and Use Committee.

## Results

### The clinical characteristics of families with ARCA

**Figure 1:**
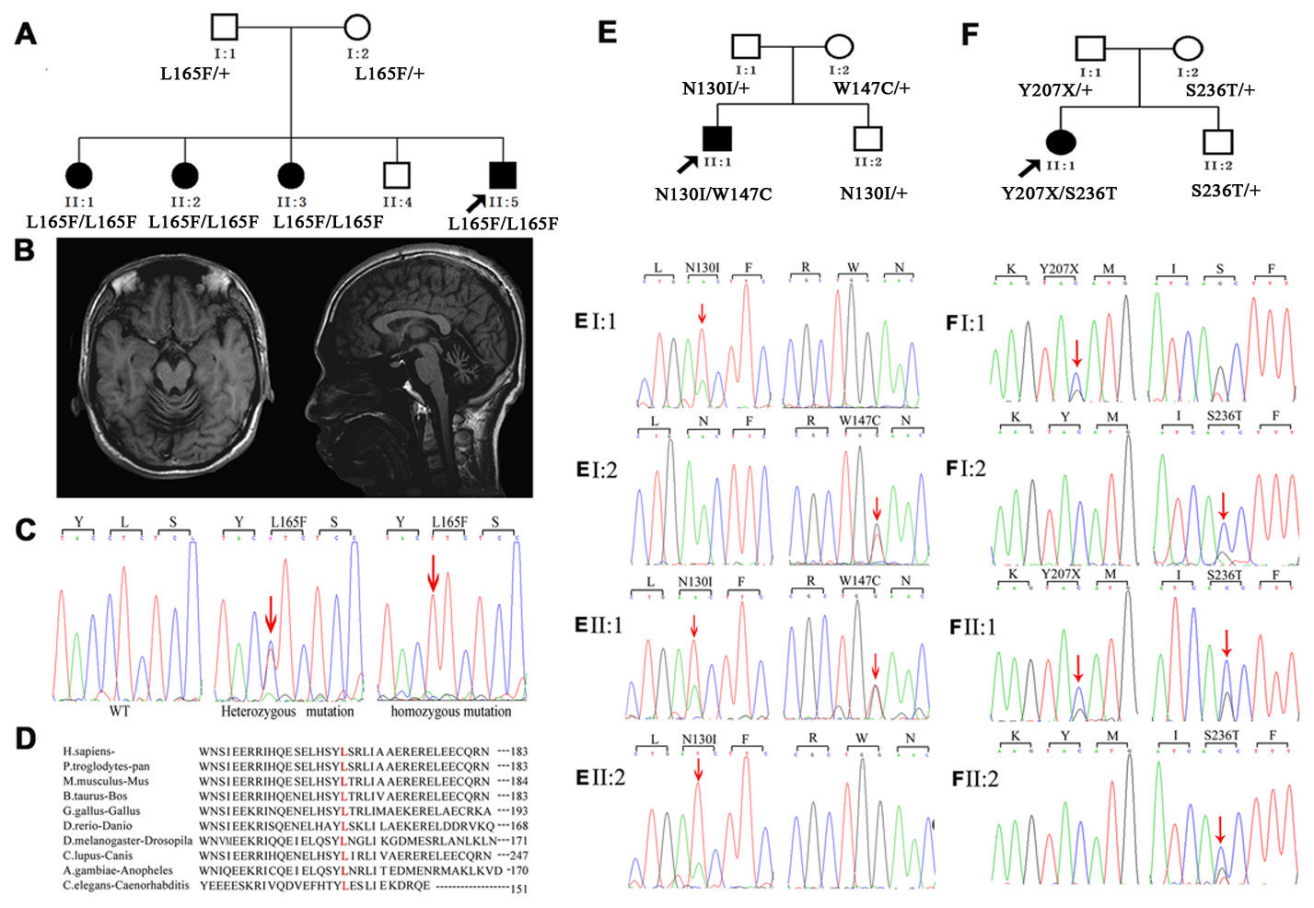
The pedigrees, brain MRIs, and CHIP mutations identified. (**A**) The pedigree of family 1 with autosomal-recessive spinocerebellar ataxia. (**B**) The brain MRI of II-5 in family 1. Panel (left): axial T1-weighted image showing atrophy of the cerebellar vermis. Panel (right): midline sagittal T1-weighted image showing cerebellar atrophy, particularly evident in the superior vermis, with enlargement of the fourth ventricle. (**C**) Sanger sequencing results of codons 164–166 in exon 1 of the CHIP gene in a WT subject (left), an individual carrying the heterozygous variant (middle), and an individual carrying the homozygous c.493C>T (p.L165F) mutation (right). (**D**) The L165F missense mutation occurred at an evolutionarily conserved amino acid (in red) in the CHIP. (**E** and **F**) The pedigrees of families 2 and 3. Sanger sequencing results of the members of these two families. The red arrows indicate the mutation sites.

 A two-generation ARCA family (family 1) with four affected individuals was recruited from the Jiangxi province in China. Another two families (family 2, 3) were all from the Hunan province in China. The diagnosis of hereditary ataxia was determined according to the criteria of Harding [18]. The proband (II:5) in famlily 1 was 34 years old, and suffered from progressive ataxia beginning at the age of 21, and presented with severe atrophy in the cerebellum as determined by brain MRI (Figure 1B). The detailed clinical features at the time of initial clinical assessment in the three families are summarized in Table 1. All patients showed features of cerebellar ataxia associated with obvious cerebellar atrophy. The latest clinical assessment on the three patients (II:2, II:3, II:5) in family 1 indicated that the ICARS and SARA scores were significantly increased (Table S2 in File S1 and Text S1). Interestingly, after ~20 years with ARCA, two patients in family 1 (patient II:2 and II:3) were found to have cognitive impairment, although they did not show obvious atrophy in the cortex and hippocampus (Table S2, S3 in File S1 and Figure S1, S2). In addition, the nerve conduction velocity, electromyogram, visual evoked response, auditory evoked potential, somatosensory evoked potential in three patients (II:2, II:3, II:5) from family 1 were partly abnormal, indicating that some pathological changes occurred in not only the central nervous system but also the peripheral nerves (Table S4 in File S1). No mutation in known hereditary ataxia-linked genes was identified in these patients. 

**Table 1:** Clinical features at the time of initial clinical assessment in the three ARCA families.

	Family 1						Family 2		Family 3
	II:1	II:2	II:3		II:5		II:1		II:1
Gender	F	F		F		M	M	F	
Age (yr)	42	39		37		34	23	25	
Disease onset (yr)	17	17		14		19	20	16	
Walking ability	w	U		U		I	I	U	
Cognitive defect	+	-		+		-	-	-	
Truncal/limb ataxia	+++/+++	++/++		++/++		++/++	±/+	++/+++	
Nystagmus	-	++		-		++	-	-	
Slow saccade	-	-		-		-	-	-	
Ophthalmoplegia	+++	-		+		-	-	-	
Dysarthria	+++	++		++		+++	±	+	
Extrapyramidal signs	-	-		-		-	-	-	
Position sense	D	D		D		D	N	N	
Tendon reflex	N	↑		↑		↑	N	↑	
Ankle tone	-	-		-		-	-	±	
Plantar responses	-	+		-		+	-	+	
ICARS	77	48		64		37	19	18	
SARA	34	15		21		15	4	5	
MMSE	7	26		17		29	29	29	
ADL	35	95		85		95	100	100	
Cerebellar atrophy on MRI	not done	severe		severe		severe	severe	severe	

Clinical signs are graded as follows: - = absent or subtle; + = mild; + + = moderate; + + + = severe; w = wheelchair; u = unilateral support; I = independent. ↑ = increased; N = normal; D = defect; ADL = Activities of Daily Living scale.

### Exome sequencing combined with linkage analysis identified carboxyl terminus of the Hsc70-interacting protein gene (CHIP) as the candidate gene

To search for the candidate gene, we performed whole-exome sequencing of the DNA from two affected individuals (II:2 and II:3) of family 1 (Figure 1A) using the Illumina Genome Analyser II platform (Table S5 in File S1). Sequencing data were then aligned to the human genome reference (UCSC hg 18 version). After calling of single-nucleotide polymorphisms and insertions or deletions, we removed non-synonymous mutations, splice acceptor and donor site mutations, and indels variants that have been reported in the dbSNP129, the ‘HapMap 8’, the SNP dataset of the 1000 Genome Project and 800 additional normal subjects (data not shown). As ARCAs are inherited in an autosomal recessive fashion, we focused on homozygous and compound heterozygous mutations. After filtering (Table 2), only the *CHIP* gene remained as common in both II:2 and II:3 subjects. Sanger sequencing indicated that the patients (II:1, 2, 3 and 5) were homozygous at c.493CT (p.L165F) of *CHIP*, whereas their parents (I:1 and I:2) and normal sibling II:4 were heterozygous (Figure 1C). These findings suggest that c.493CT (p.L165F) of *CHIP* completely co-segregated with the disease phenotype within family 1. The p.L165F variant was located in a highly conserved position of CHIP (Figure 1D), however, it was not identified in any of the 500 unaffected controls matched for geographical ancestry. 

**Table 2:** Identification of the causative gene for ARCA from two patients by whole-exome sequencing.

Filter	II2	II2 (homozygote)	II3	II3 (homozygote)	II2 + II3	II2 + II3 (homozygote)	II2 + II3 (compound heterozygote)
Number of NS/SS/ Indel	7277	3393	7391	3485	7645	3557	2673
Number of NS/SS/ Indel after Filter 1	1892	508	1927	532	874	344	69
Number of NS/SS/ Indel after Filter 2	1028	328	1048	336	566	129	46
Number of NS/SS/ Indel after Filter 3	368	161	389	163	399	48	22
Number of NS/SS/ Indel after Filter 4	82	41	89	43	29	1	0

“Shared genes” indicated the gene mutations occurred in all three samples. Columns show the effect of requiring that non-synonymous/splice acceptor and donor site/insertions or deletions (NS/SS/Indel) variants be observed in each affected individual (Columns 2-5) or 2 shared affected individuals (Columns 6-7). Homozygote or compound heterozygote in the brackets indicate that we only focus on the shared homozygote or compound heterozygote in the same causal gene. In the step of Filter 1, we first removed the NS/SS/Indel variants reported in the dbSNP129. Then, the NS/SS/Indel variants reported in the 1000 genome project were further removed in Filter 2. Consequently, the NS/SS/Indel variants reported in the eight previously exome-sequenced HapMap samples (‘HapMap 8’) and YH database were removed in Filter 3. At last, the NS/SS/Indel variants reported in house database exome normal control cases (unpublished data) were removed in Filter 4.

To exclude the possible contribution of CNVs, which cannot be determined by exome sequencing, we surveyed the whole genomes of seven subjects (I:1, I:2, and II:1-5) in family 1 for CNVs using the Illumina HumanHap660 Bead Chip. No CNVs co-segregated with the phenotype. Further, we carried out linkage analysis of whole-genome SNPs. Interestingly, we mapped a linkage to a minimal candidate region of 8.55cM on chromosome 16p13.3 flanked by SNP markers rs11248850 and rs1218762, where the *CHIP* gene is located. The multi-point logarithm of the odds score was ascertained as 1.917 (Table S6 in File S1). 

### Mutations in the CHIP gene

**Figure 2:**
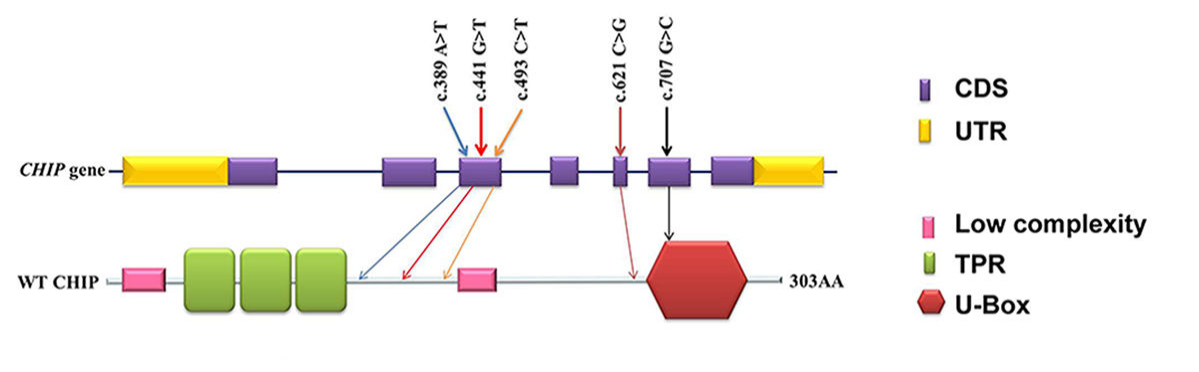
Genomic organization of the human CHIP gene and the domain structure of the CHIP protein. The CHIP gene consists of seven exons. The CHIP protein has two key domains: the TPR domains and the one U-box domain. The five mutations identified in CHIP are indicated with arrows. Three mutations [c.493 CγT (p.L165F); c.389A>T (p.N130I); c.441G>T (p.W147C); c.707G>C (p.S236T)] are were located between the third TPR domain and the second low complexity segment. The c.621C>G (p.Y207X) mutation encodes a truncated protein without a U-box domain and the S236T mutation is located in the U-box domain.

CHIP, also known as the *STIP1* homology and U box-containing protein 1 (STUB1), is a multifunctional E3 ubiquitin ligase. As shown in Figure 2, the *CHIP* gene consists of seven exons, and its protein harbors three domains: an N-terminal three-tetratricopeptide repeat domain, a highly charged middle domain, and a carboxyl-terminal U-box domain [19] (Figure 2). The TPR domain of CHIP serves as protein-protein interaction domain that has traditionally been thought to mediate interactions with heat shock proteins [20], while the U-box domain of CHIP acts as an ubiquitin ligase [21]. The amino acid sequence of human CHIP has 97% identity and 98% similarity with the mouse Chip. The c.493CT (p.L165F) mutation we found in family 1 was a missense mutation in the third exon of *CHIP*. 

To identify additional deleterious variants among other families with ARCA, we performed Sanger sequencing to screen the exons and flanking introns of the *CHIP* gene in an additional 36 families with histories of ARCA and 196 sporadic individuals affected with ataxia; individuals with the common cerebellar ataxia-linked genes were excluded. *CHIP* mutations were found in two additional ARCA families. One compound heterozygous mutation [c.389AT (p.N130I) and c.441GT (p.W147C)] was identified in family 2 (Figure 1E), and another compound heterozygous mutation [c.621CG (p.Y207X) and c.707GC (p. S236T)] was identified in family 3 (Figure 1F). The c.621CG (p.Y207X) mutation is a non-sense mutation that was found in the fifth exon of *CHIP*; this mutation substitutes the codon for Y207 (TAC) with a stop codon (TAG) and generates a truncated protein with only 206 amino acids. The other mutant forms including c.389AT (p.N130I), c.441GT (p.W147C), and c.707GC (p. S236T) are missense mutations. These mutations completely co-segregated with the phenotype in these two families and were not detected in 500 controls who were matched for geographic origin. All the five variants (c.389AT, c.441GT, c.493CT, c.621CG and c.707GC) were predicted to be probably damaging (Table S7 in File S1).

### The distribution of CHIP in the mouse brain

**Figure 3:**
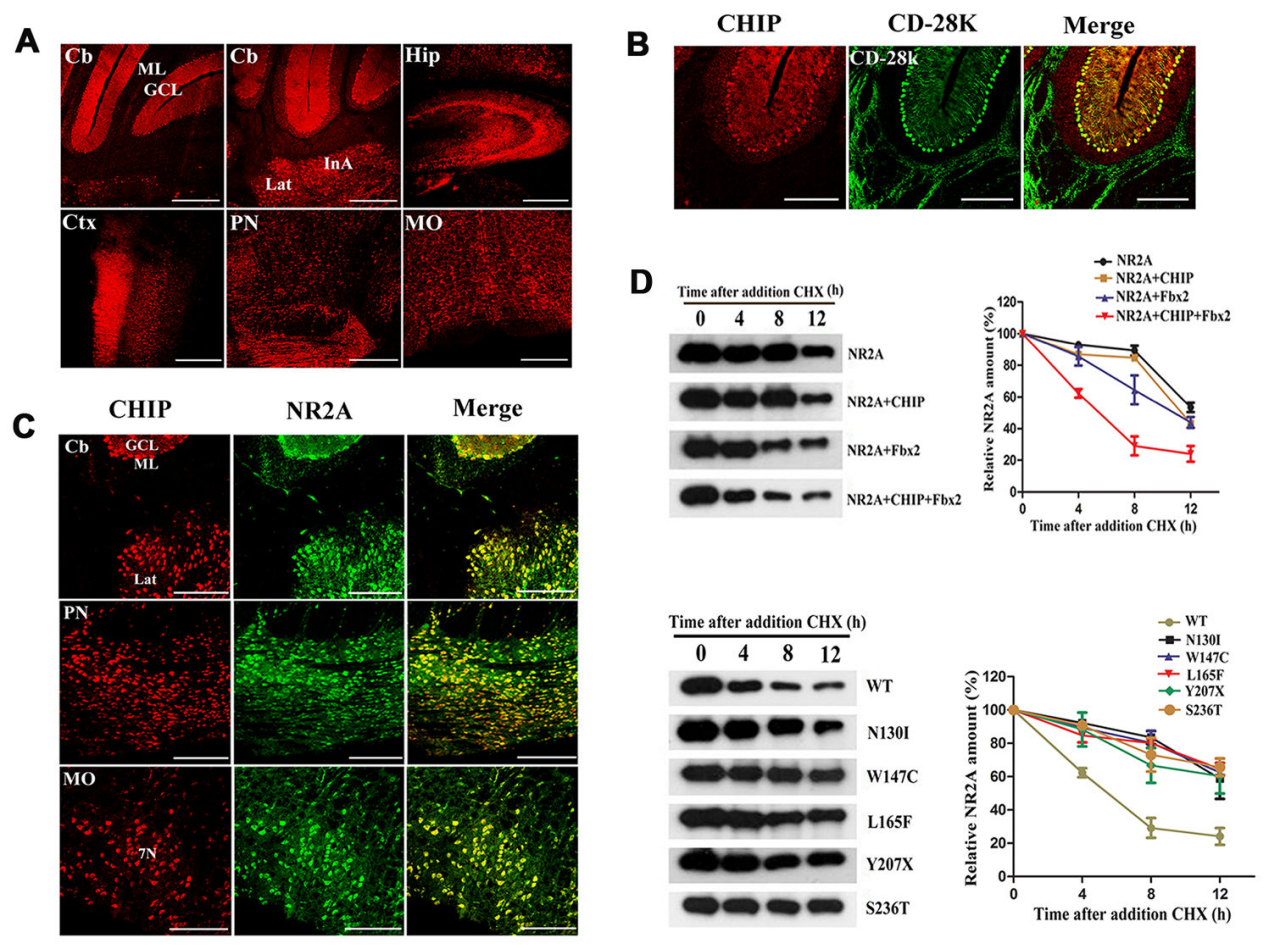
Expression of CHIP in the mouse brain and the effect of ARCA-associcated mutations on its ability to promote the degradation of NR2A. (**A**) The expression of CHIP in the cerebellum (Cb), hippocampus (Hip), cerebral cortex (Ctx), pons (PN) and medulla oblongata (MO) of mouse brain as analyzed with immunohistochemistry. (**B**) Co-localization of CHIP (red) and calcium-binding protein calbindin D-28K (green) in Purkinje cells. (**C**) Co-localization of CHIP (red) and NR2A (green) in Cb, PN and MO. (**D**) Coexpression of flag-tagged WT, but not ARCA-associated CHIP mutants (CHIP^N130I^, CHIP^W147C^, CHIP^L165F^, CHIP^Y207X^, CHIP^S236T^), with HA-Fbx2 promoted the degradation of NR2A. Expression vectors for CHIP, Fbx2 and NR2A were transfected into Human Embryonic Kidney 293 cells. At 36 h after transfection, cells were treated with cycloheximide (CHX, 100 μg/ml) and chased for different time periods. NR2A was detected with western blot using the Myc antibody. Quantitative analysis was performed using NIH ImageJ analysis software. Values represent the mean ± S.D. of three independent experiments.

Using immunofluorescence, we showed that CHIP was highly expressed in the cerebellum, pons, medulla oblongata, hippocampus and cerebral cortex (Figure 3A). Moreover, we demonstrated that CHIP co-localized with the calcium-binding protein calbindin D-28K in Purkinje cells (Figure 3B), which is an essential determinant of normal motor coordination and sensory integration in the cerebellum [22]. 

### Characterization of the activity of mutant CHIPs

N-methyl-D-aspartate receptors (NMDARs) are mainly composed of a structural NR1 subunit and a NR2 subunit (NR2A-D), which modulate the biophysical properties of the NMDARs [23-25]. Nelson et al reported that CHIP functions with Fbx2 to promote the ubiquitination and degradation of NMDARs [26]. Indeed, the levels of NR2B (the embryonic counterpart of NR2A) are significantly elevated in cultured cortical neurons from embryonic CHIP -/- mice [26]. NMDARs play a fundamental role in excitatory neurotransmission and cerebellar-dependent motor coordination [27]; thus the alterations of NMDAR function in CHIP knockout mice may underlie the pathogenesis of ataxia.

To characterize the ubiquitin ligase activity of mutant CHIPs, we chose NR2A as a test substrate. In the mouse brain, we observed that CHIP co-localized with NR2A in the cerebellum, pons, and medulla oblongata (Figure 3C). Coexpression of WT CHIP and Fbx2 significantly increased the degradation of NR2A, whereas WT CHIP or Fbx2 alone did not produce significant effects on the degradation of NR2A. In contrast, none of the ARCA-associated CHIP mutants effectively promoted the degradation of NR2A (Figure 3D).

## Discussion

Previous studies have shown that impairments of the ubiquitin proteasome system (UPS) are associated with the formation of inclusions in neurodegenerative diseases [28]. Mutations in the gene involved in the UPS pathway have been reported in neurodegenerative diseases, such as Parkin, an E3 ligase, in Parkinson’s disease and UBQLN2, a ubiquitin-like protein, in dominant X-linked juvenile and adult onset amyotrophic lateral sclerosis [29,30]. Moreover, mutations in different E3 ubiquitin ligase genes have been identified in several neurodegenerative diseases, such as Sacsin in the autosomal recessive spastic ataxia of Charlevoix-Saguenay, Gigaxonin in Giant axonal neuropathy and Malin in Lafora disease [31-33]. Recently, Margolin et al identified mutations of RNF216 and OTUD4, which encode an E3 ubiquitin ligase and a deubiquitinase respectively, in a patient with ataxia and hypogonadotropism [34]. These findings suggest that disordered ubiquitination is involved in a broad spectrum of neurodegenerative disorders. Here, we identified *CHIP*, which encodes a U-box containing E3 ubiquitin ligase, as the causative gene in patients with ARCA.

CHIP has been implicated in several neurodegenerative disorders that are characterized by protein misfolding and aggregation. And CHIP was demonstrated to regulate degradation of expanded ataxin-1, ataxin-3, huntingtin and androgen receptor [35-39]. Parkin, α-Synuclein and LARRK2 associated with familial PD have been shown to be clients of CHIP and CHIP immunoreactivity has been detected in Lewy bodies in PD [40-42]. CHIP immunoreactivity has also been detected in the ataxin-1 nuclear inclusions (NIs) that are present in the brains of spinocerebellar ataxia type-1 patients [35]. Moreover, CHIP levels are increased in the brains of patients with Alzheimer's disease and CHIP levels are inversely proportional to the amount of accumulated tau protein in the brains of Alzheimer’s disease patients [36]. Previous studies have also shown that CHIP-deficient mice exhibit profound dysbasia, low body weights and premature aging phenotypes [36,43]. Here, we provide direct evidence that mutations in *CHIP* are associated with ARCA. 

CHIP is highly expressed in the brains, striated muscle tissue and pancreases of humans [19]. The high expression of CHIP in tissues with high metabolic activities are consistent with CHIP’s crucial role in protein quality control, and this role is, perhaps, mediated by the promotion of degradation of damaged proteins or the enabling of activation of the heat shock response [44]. CHIP is constitutively localized in the cytoplasm under normal condition, but can be translocated into the nucleus during stress [19]. In this study, CHIP was found to be highly expressed in the cerebellum, pons, medulla oblongata, hippocampus and cerebral cortex of mice. In the cerebellum, CHIP was co-localized in Purkinje cells with calcium-binding protein calbindin D-28K, which plays an important role in maintaining the balance of calcium in neurons [45]. 

CHIP is a multifunctional E3 ubiquitin ligase, that attaches ubiquitin to protein substrates, and marks them for UPS. It has been reported that the interaction of CHIP with Fbx2 can facilitate the ubiquitination and degradation of Fbx2-bound proteins including NR2A and CHIP-deficient neurons have increased NR2B levels [26]. In this study, we chose to investigate the ability of CHIP to facilitate the degradation of NR2A. We demonstrated that CHIP co-localized with NR2A in the mouse cerebellum, pons, and medulla oblongata. Fbx2 interacts with the three-tetratricopeptide repeat domain of CHIP, which is intact in the five ARCA associated CHIP mutants. Indeed, all the ARCA associated mutations did not interfere with the formation of CHIP-Fbx2-NR2A complex (Figure S3). Nevertheless, co-expression of WT, but not ARCA-associcated mutant CHIP, with Fbx2 promoted the degradation of NR2A. Our data suggest that ARCA-associated CHIP mutations are deleterious and that the inability to degrade NMDARs in neurons may be an underlying mechanism of the development of ARCA. 

In conclusion, using a combination of whole-exome sequencing and linkage analysis, we have identified homozygous or compound heterozygous mutations in *CHIP* led to ARCA. The impairment of disease-associated mutant CHIPs to promote the degradation of NR2A may underlie the pathogenesis of ataxia. 

## Supporting Information

Figure S1 **Brain MRI of patient II-2 in Family 1.** (**A**) Axial T1-weighted image showing atrophy of the cerebellar vermis. (**B**) Sagittal T1-weighted image showing cerebellar atrophy, particularly evident in the superior vermis, with enlargement of the fourth ventricle. (**C**) Coronal T1-weighted image showing no atrophy of the hippocampus and cerebrum.
(TIF)

Figure S2 **Brain MRI of patient II-3 in Family 1.** (A) Axial T1-weighted image showing atrophy of the cerebellar vermis. (B) Sagittal T1-weighted image showing cerebellar atrophy, particularly evident in the superior vermis, with enlargement of the fourth ventricle. (C) Coronal T1-weighted image showing no atrophy of the hippocampus and cerebrum.
(TIF)

Figure S3 **Interactions of wide-type CHIP and its mutants with Fbx2 and NR2A.** Expression vectors for CHIP, Fbx2 and NR2A were transfected into HEK293 cells. At 36h after transfection, Cell lysates were immunoprecipitated (IP) with anti-Flag antibody and bound proteins were revealed by immunoblot (IB) with anti-myc and anti-HA antibodies.
(TIF)

File S1 **Supporting tables**.
Table S1, The primers used for cDNA amplification of CHIP. Table S2, The latest clinical features in the affected members of the three families (updated in Aug. 2013). Table S3, Intelligence test scores of three patients from family 1 by the WAIS-RC. Table S4, Electrodiagnostic studies performed on three patients of family 1. Table S5, Summary of original Exome sequencing data. Table S6, Multi-point LOD scores between the disease locus and SNP polymorphism markers in family 1. Table S7, Prediction of the functional effects of mutantion in *CHIP*.
(DOCX)

Text S1 **Additional clinical information regarding the ARCA cases in our study.**
(DOCX)
