# Supplementary figures and images for: Identification of CHIP as a Novel Causative Gene for Autosomal Recessive Cerebellar Ataxia
Source: PLoS One. 2013 Dec 2;8(12):e81884. doi: 10.1371/journal.pone.0081884 (PMC3846781; doi:10.1371/journal.pone.0081884)

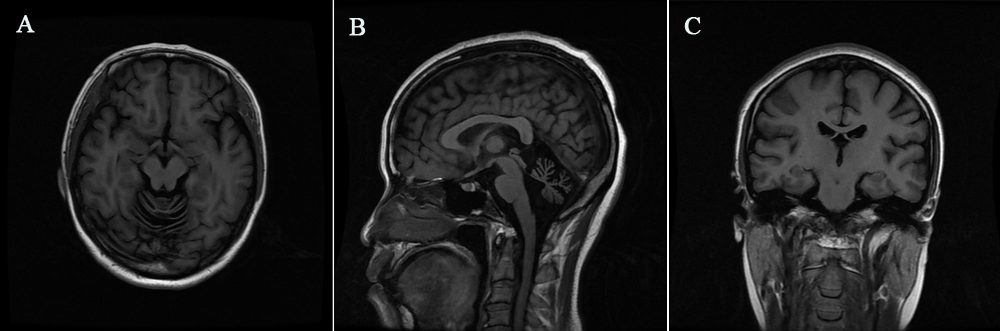

Supplement: Figure S1 — Brain MRI of patient II-2 in Family 1. (A) Axial T1-weighted image showing atrophy of the cerebellar vermis. (B) Sagittal T1-weighted image showing cerebellar atrophy, particularly evident in the superior vermis, with enlargement of the fourth ventricle. (C) Coronal T1-weighted image showing no atrophy of the hippocampus and cerebrum. (TIF) [file pone.0081884.s001.tif]

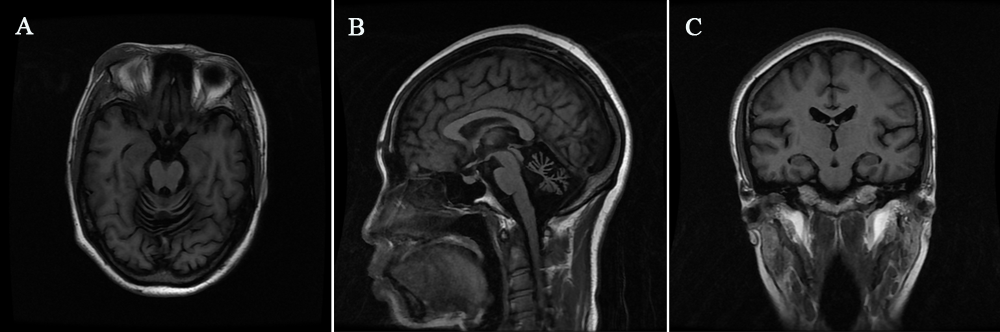

Supplement: Figure S2 — Brain MRI of patient II-3 in Family 1. (A) Axial T1-weighted image showing atrophy of the cerebellar vermis. (B) Sagittal T1-weighted image showing cerebellar atrophy, particularly evident in the superior vermis, with enlargement of the fourth ventricle. (C) Coronal T1-weighted image showing no atrophy of the hippocampus and cerebrum. (TIF) [file pone.0081884.s002.tif]

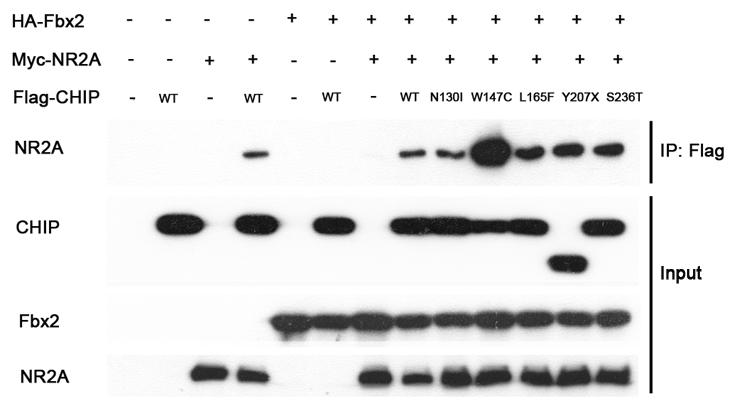

Supplement: Figure S3 — Interactions of wide-type CHIP and its mutants with Fbx2 and NR2A. Expression vectors for CHIP, Fbx2 and NR2A were transfected into HEK293 cells. At 36h after transfection, Cell lysates were immunoprecipitated (IP) with anti-Flag antibody and bound proteins were revealed by immunoblot (IB) with anti-myc and anti-HA antibodies. (TIF) [file pone.0081884.s003.tif]
