# Supplementary material for: Identification of CHIP as a Novel Causative Gene for Autosomal Recessive Cerebellar Ataxia
Source: PLoS One. 2013 Dec 2;8(12):e81884. doi: 10.1371/journal.pone.0081884 (PMC3846781; doi:10.1371/journal.pone.0081884)
Supplement: File S1 — Supporting tables. Table S1, The primers used for cDNA amplification of CHIP. Table S2, The latest clinical features in the affected members of the three families (updated in Aug. 2013). Table S3, Intelligence test scores of three patients from family 1 by the WAIS-RC. Table S4, Electrodiagnostic studies performed on three patients of family 1. Table S5, Summary of original Exome sequencing data. Table S6, Multi-point LOD scores between the disease locus and SNP polymorphism markers in family 1. Table S7, Prediction of the functional effects of mutantion in CHIP. (DOCX) [file pone.0081884.s004.docx]

**Supporting information**

**Table S1.** **The** **primers used for cDNA amplification of** **CHIP.**

| wide-type or mutant CHIP | primers |
| --- | --- |
| W1: wide-type | F: atgaagggcaaggaggagaa |
| W2: wide-type | R: agggcagggaacctcagtag |
| M1: L165F | F: cctacttctccaggctcatt |
| M2: L165F | R: agtgcagctcgctct |
| M3: N130A- F | F: cggctgatcttcggggacgacat |
| M4: N130A-R | R: ctgctccttggccaggctgta |
| M5: W147C-F | F: aagcgctgtaacagcatt |
| M6: W147C-R | R: cttcttcgcgattcgaag |
| M7: Y207-F | F: aagtagatggcggacatggac |
| M8: Y207-R | R: gtcgtgcttggcctcaat |
| M9: S236T-F | F: acctttgagctgatgcgggag |
| M10: S236T-R | R: gatcttgccacacaggta |

**Table S2.** **The latest clinical features** **in the affected members of the three families (updated in Aug. 2013).**

|  | Family 1 | | | | Family 2 | Family 3 |
| --- | --- | --- | --- | --- | --- | --- |
|  | II:1 | II:2 | II:3 | II:5 | II:1 | II:1 |
| Gender | F | F | F | M | M | F |
| Age (yr) | 44 | 41 | 39 | 36 | 25 | 27 |
| Disease onset (yr) | 17 | 17 | 14 | 19 | 20 | 16 |
| Walking ability | w | U | U | U | I | U |
| Cognitive defect | + | + | + | + | - | - |
| Truncal/limb ataxia | +++/+++ | ++/++ | ++/++ | ++/++ | ±/+ | ++/+++ |
| Nystagmus | - | ++ | ++ | ++ | - | - |
| Saccade slowing | - | - | - | - | - | - |
| Ophthalmoplegia | +++ | - | + | - | - | - |
| Dysarthria | +++ | ++ | ++ | +++ | ± | + |
| Extrapyramidal signs | - | - | - | - | - | - |
| Position sense | D | D | D | D | N | N |
| Tendon reflex | N | ↑ | ↑ | ↑ | N | ↑ |
| Ankle tone | - | - | - | - | - | ± |
| Plantar responses | - | + | - | + | - | + |
| ICARS | 80 | 61 | 72 | 57 | 26 | 36 |
| SARA  MMSE | 35  6 | 21  18 | 24  10 | 20  29 | 8  29 | 15  29 |
| WAIS-RC | not done | 53 | 33 | 82 | not done | not done |
| ADL | 25 | 30 | 40 | 30 | 95 | 85 |
| Cerebellar atrophy on MRI | not done | severe | severe | severe | severe | severe |

Clinical signs are graded as follows: - = absent or subtle; + = mild; + + = moderate; + + + = severe; w = wheelchair; u = unilateral support; I =independent; N = normal; D = defect; WAIS-RC = Wechsler Adult Intelligence Scale (the Revised Chinese version); ADL = Activities of Daily Living scale. The cognitive abilities of these patients were evaluated by at least one cognitive scale, including the Mini-Mental State Examination (MMSE) ([Folstein *et al.*, 1975](#_ENREF_1)) and the Chinese Revised Wechsler Adult Intelligence Scale (WAIS-RC) (Gong YX. 1992).

**Table S3.** **Intelligence test scores of three patients from family 1 by** **the WAIS-RC.**

| **Family 1** | **FSIQ** | **PIQ** | **VIQ** |
| --- | --- | --- | --- |
| **Patient II:2** | **53** | **49** | **62** |
| **Patient II:3** | **33** | **37** | **37** |
| **Patient II:5** | **82** | **74** | **92** |

Global cognitive functioning (Full Scale IQ) was assessed using the Chinese Revised Wechsler Abbreviated Scale of Intelligence

(WASI-RC) for both verbal (Verbal IQ [VIQ]) and nonverbal ability (or Performance IQ [PIQ]) (Gong XY. 1992; Wechsler, 1999).

**Table S4. Electrodiagnostic studies performed on three patients of Family 1.**

|  |  | | | | **Family 1** | | | | |  | | | |
| --- | --- | --- | --- | --- | --- | --- | --- | --- | --- | --- | --- | --- | --- |
|  | **II:2** | | | | **II:3** | | | | | **II:5** | | | |
| **Nerve Conduction study** | Right/ Left | | | | Right/ Left | | | | | Right/ Left | | | |
| Motor nerve conduction | **median** | **ulnar** | **tibial** | **sural** | **median** | **ulnar** | **tibial** | | **sural** | **median** | **ulnar** | **tibial** | **sural** |
| Lantency (ms) | N/ N | N/ N | N/ N | N/ N | P/ N | N/ N | N/ N | | N/ N | N/ N | N/ N | P/ P | P/ P |
| Amplitude (mV) | N/ N | N/ N | Low/ Low | N/ N | Low/N | N/ N | N/ N | | N/ N | N/ N | N/ Low | N/ N | Low/N |
| NCV (m/s) | Slow/ N | N/ N | N/ N | N/ N | N/ N | N/ N | Slow/ Slow | | N/ N | N/ N | N/ Slow | Slow/ Slow | Slow/ N |
| Sensory nerve conduction | **median** | **ulnar** | **tibial** | **sural** | **median** | **ulnar** | **tibial** | | **sural** | **median** | **ulnar** | **tibial** | **sural** |
| Lantency (ms) | N/ N | N/ N | N/ N | N/ N | N/ Slow | N/ N | N/ N | | N/ N | N/ N | N/ N | N/ N | N/ N |
| Amplitude (uV) | N/ N | N/ N | N/ N | N/ N | Low/ N | Low/ Low | N/ N | | N/ N | N/ N | Low/ Low | low | Low/ Low |
| NCV (m/s) | Slow/ N | N/ N | N/ N | N/ N | N/ Low | N/ N | N/ N | | N/ N | N/ N | N/ N | Slow | N/ N |
| F-wave  Latency (ms)  Occurrence | Right  N  Ankle: 18% | | Left  N  Ankle: 100% | | Right  N  Wrist: 13% | | Left  N  Ankle: 100% | | | Right  N  Ankle: 100% | | Left  N  Ankle: 100% | |
| **Electromography** | N | | N | | N | | N | | | N | | N | |
| **Visual Evoked Response** | **Right** | | **Left** | | **Right** | | **Left** | | | **Right** | | **Left** | |
| Latency of P100 in visual half field | prolonged | | normal | | normal | | prolonged | | | normal | | normal | |
| Amplitude of P100 in visual half field | Low | | Low | | Low | | Low | | | Low | | Low | |
| Latency of P100 in visual whole field | prolonged | | normal | | prolonged | | nromal | | | normal | | normal | |
| Amplitude of P100 in visual whole field | Low | | Low | | Low | | Low | | | Low | | Low | |
| **Auditory Evoked Potential** | **Right** | | **Left** | | **Right** | | **Left** | | | **Right** | | **Left** | |
| Latencies (I, III and V) | prolonged | | normal | | normal | | normal | | | normal | | prolonged | |
| Amplitude (I, III and V) | Low | | Low | | Low | | Low | | | Low | | Low | |
| Peak interval of I-III | normal | | normal | | normal | | normal | | | normal | | prolonged | |
| Peak interval of III-V | normal | | normal | | normal | | normal | | | normal | | normal | |
| **Somatosensory Evoked Potential** | **Right** | | **Left** | | **Right** | | **Left** | | | **Right** | | **Left** | |
| median nerve |  | | | | | | | | | | | | |
| P15, N20, P25, N35, N20-N13, N20-N9 | disappeared | | disappeared | | disappeared | | disappeared | | | disappeared | | disappeared | |
| N13, N9 and N13-N9 | N | | N | | N | | N | | | N | | N | |
| tibial nerve |  | | | | | | | | | | | | |
| P40, N45, P40-LP, P40-PF | disappeared | | disappeared | | disappeared | | | disappeared | | disappeared | | disappeared | |
| LP, PF and LP-PF | N | | N | | N | | | N | | N | | N | |

**Note:** ‘N’ means normal; ‘P’ means prolonged.

**Table S****5.** **Summary of original Exome sequencing data.**

| Sample | Bases (MB) | Map Bases  (MB) | Map Bases  Rate (%) | Exon Map Bases  (MB) | Exon Map  Bases Rate (%) | Exon  Length  (MB) | Covered  Length  (MB) | Coverage (%) | Mean  Depth | Mode  Depth |
| --- | --- | --- | --- | --- | --- | --- | --- | --- | --- | --- |
| II2 | 5782.88 | 4918.17 | 85.05 | 2376.96 | 48.33 | 37.62 | 36.78 | 97.66 | 55.76 | 38.45 |
| II3 | 6137.07 | 4851.29 | 79.05 | 2234.19 | 46.05 | 37.62 | 36.71 | 97.58 | 54.44 | 40.63 |
| Mean | 5959.98 | 4884.73 | 82.05 | 2305.58 | 47.19 | 37.62 | 36.75 | 97.62 | 55.10 | 39.54 |

**Table S6.** **Multi-point LOD scores between the disease locus and SNP polymorphism markers in Family 1.**

| Locus | Marker | Position from Peter(cM) | LOD |
| --- | --- | --- | --- |
| 16p13.3 | rs11248850 | 1.14 | 1.917 |
| 16p13.3 | rs8051485 | 1.30 | 1.917 |
| 16p13.3 | rs3752496 | 2.53 | 1.917 |
| 16p13.3 | rs12448639 | 2.78 | 1.917 |
| 16p13.3 | rs556179 | 3.04 | 1.917 |
| 16p13.3 | rs4984707 | 3.20 | 1.917 |
| 16p13.3 | rs4984727 | 3.34 | 1.917 |
| 16p13.3 | rs11248851 | 3.67 | 1.917 |
| 16p13.3 | rs1033466 | 4.88 | 1.917 |
| 16p13.3 | rs2437732 | 5.77 | 1.917 |
| 16p13.3 | rs2252523 | 6.26 | 1.917 |
| 16p13.3 | rs8057913 | 6.47 | 1.917 |
| 16p13.3 | rs26866 | 7.00 | 1.917 |
| 16p13.3 | rs26845 | 7.13 | 1.917 |
| 16p13.3 | rs4785919 | 8.74 | 1.917 |
| 16p13.3 | rs7194018 | 8.76 | 1.917 |
| 16p13.3 | rs6501170 | 9.63 | 1.917 |
| 16p13.3 | rs1218762 | 9.69 | -3.343 |

LOD＝logarithm of odds. LOD scores were calculated under an autosomal recessive mode of inheritance.

**Table S7.** **Prediction of the functional effects of mutantion in *CHIP.***

| Mutation in *CHIP* | Mutation taster | SIFT | polyphen-2 |
| --- | --- | --- | --- |
| c. 389 A>T (N130I) | disease causing | probably damaging | probably damaging |
| c. 441 G>T (W147C) | disease causing | probably damaging | probably damaging |
| c. 493 C>T (L165F) | disease causing | probably damaging | probably damaging |
| c. 621 C>G (Y207X) | disease causing | probably damaging | probably damaging |
| c. 4707 G>C (S236T) | disease causing | probably damaging | probably damaging |

Note: Mutation taster (www.mutationtaster.org/‎), SIFT (sift.jcvi.org/) and polyphen-2 (genetics.bwh.harvard.edu/pph2/‎).

Reference

Folstein MF, Folstein SE, McHugh PR. "Mini-mental state". A practical method for grading the cognitive state of patients for the clinician. J Psychiatr Res. 1975;12(3):189-98.

Gong Y X. Manual of Wechsler Adult Intelligence Scale-Chinese version. Changsha: Chinese Map Press. 1992
