# Supplementary material for: Identification of CHIP as a Novel Causative Gene for Autosomal Recessive Cerebellar Ataxia
Source: PLoS One. 2013 Dec 2;8(12):e81884. doi: 10.1371/journal.pone.0081884 (PMC3846781; doi:10.1371/journal.pone.0081884)
Supplement: Text S1 — Additional clinical information regarding the ARCA cases in our study. (DOCX) [file pone.0081884.s005.docx]

**Supporting information**

**Additional clinical information regarding the ARCA cases of** **family 1.**

The latest clinical features of family 1 are summarized in Table S2. The age of onset ranged from 14–21 years and the disease duration varied from 15–27 years. Walking difficulty and cerebellar dysarthria were early features in all individuals, and incoordination of the upper extremities developed relatively slowly. The ICARS and SARA scores clearly showed that clinical severity grossly correlated with the duration of the disease. After disease durations of ~20 years, most patients required unilateral support or wheelchairs outdoors, and these patients were found to have cognitive impairments through neurological examination, which had multiple cognitive domains impairment, including comprehension, calculation, orientation and language (Table S3). The cognitive abilities of these patients were evaluated by at least one cognitive scale, including the Mini-Mental State Examination (MMSE) ([Folstein *et al.*, 1975](#_ENREF_1)) and the Chinese Revised Wechsler Adult Intelligence Scale (WAIS-RC) (Gong YX. 1992). The proband in family 1 (patient II:5) was found to have slight intelligent impairment examined by WAIS-RC while he got 29 scores assessed by MMSE (Table S3). Levels of blood glucose, blood electrolytes, serum follicle stimulating hormone, luteinizing hormone, estradiol, prolactin, progesterone, testosterone, vitamin B12, vitamin E and folic acid, as well as electrocardiogram of these patients were all normal. Nerve conduction velocity (NCV), electromyogram (EMG), visual evoked response (VEP), auditory evoked potential (AEP), somatosensory evoked potential (SEP) were carried on three patients (II:2, II:3, II:5) from family 1, which were shown in Table S4. The video materials of the patients (II:2, II:3 and II:5) of the primary family (family 1) were shown in supplementary data.

**Additional clinical information regarding the ARCA cases of family 2 and 3.**

**Family 2- II-1.** This patient had a normal childhood and began showing difficulty walking at the age of 20 years old. He could walk independently but with a stumbling gait. The symptoms of this patient were relatively mild and progressed slowly. He exhibited no nystagmus, slow saccade, sensory abnormality and cognitive problems.

**Family 3- II-1.** This patient had a normal childhood. She began to show difficulty walking at the age of 16 years old. This patient had poor dexterity and coordination in all extremities, and significantly more so in her legs. She required unilateral support while walking. She did not show cognitive dysfunction, nystagmus, slow saccade and sensory abnormality.
